# When working memory works for our goals

**DOI:** 10.7554/eLife.106869

**Published:** 2025-04-24

**Authors:** Jacob A Miller

**Affiliations:** 1 Department of Psychology, University of Miami Miami United States

**Keywords:** working memory, cognition, frontal cortex, visual cortex, fMRI, recurrent neural network, Human

## Abstract

When navigating environments with changing rules, human brain circuits flexibly adapt how and where we retain information to help us achieve our immediate goals.

**Related research article** Shao Z, Zhang M, Yu Q. 2025. Stimulus representation in human frontal cortex supports flexible control in working memory. *eLife* **13**:RP100287. doi: 10.7554/eLife.100287.

Driving through the streets of a bustling city, you suddenly decide to pull up to park. If you want to avoid a ticket, this is not a simple cognitive task. You will need to remember information splayed across street signs you have now long driven by: which side of the street are you allowed to park on? At what times? On which days?

Information that we cannot see anymore can be stored for seconds in our working memory (Repovs and Baddeley, 2006). Rather than being a simple ‘mental sketchpad’ of past events, however, working memory guides our future behavior and actions (van Ede and Nobre, 2023). Exactly how and where this takes place remain fundamental questions of cognitive neuroscience. In fact, seemingly contradictory theories and evidence exist on the various neural circuits that can participate in this process.

For one, visual areas that process ongoing sensory signals at the back of the brain can be ‘recruited’ to hold recent information in working memory (D’Esposito and Postle, 2015; Serences, 2016). Yet, under some circumstances, specific working memories are detectable in the frontal cortex, a region at the opposite side of the brain that is involved in high-level cognitive processes (Christophel et al., 2017; Ester et al., 2015).

The brain areas and mechanisms that are recruited to support working memory likely differ based on the type of information that needs to be remembered, and what it will be used for. In laboratory settings, the choice of experimental design – such as the brain recording method, training or task timing – is also likely to have an impact. Now, in eLife, Zhujun Shao, Mengya Zhang and Qing Yu (from the Chinese Academy of Sciences, Shanghai and the University of Chinese Academy of Sciences, Beijing) report that the human frontal cortex flexibly adapts its role for working memory storage depending on the task at hand (Shao et al., 2025).

To test this idea, Shao et al. designed a complex working memory experiment in which goals, rules and cognitive demands changed quickly between trials (Figure 1). It relied on stimuli consisting of simple lines slanted at different, pre-established angles. In ‘maintenance’ trials, participants were asked which of two lines matched the one they were tasked to remember at the start of the trial; ‘categorization’ trials, however, were more complex. Participants had to recall a previously learnt rule that allowed them to assign the stimulus to one out of two categories based on its orientation. Crucially, categorization or maintenance trials were randomly ordered, and the categorization rule changed every nine blocks.

**Figure 1.**
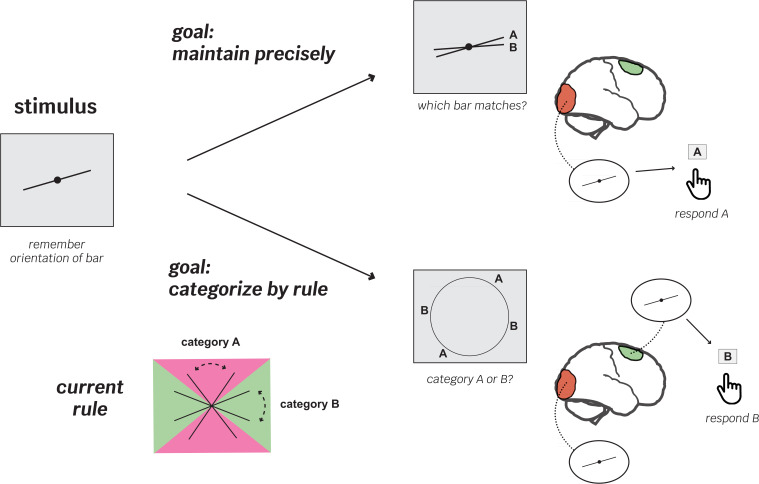
Flexible recruitment of the frontal cortex helps support working memory in rule-based recall tasks. The experiments by Shao et al. aimed to explore how different brain areas are recruited in working memory processes depending on the nature of the task at hand. They involved participants observing two oriented lines and being asked to remember one of them (stimulus). First, the volunteers learned categorization rules (bottom left); depending on its orientation, a line could belong to either ‘category A’ (pink area) or ‘B’ (green area). After completing this training, the participants moved on to the trial experiments, which mixed ‘maintenance’ trials (where participants were asked which of two newly shown lines matched the one they had been asked to remember; top) with ‘categorization’ trials (which relied on the previously learned rule; bottom). In the first experiment, categorization rules switched among two possibilities after blocks of nine trials. The team recorded brain activity throughout the experiments using functional magnetic resonance imaging. On trials with precise maintenance demands, patterns of brain activity in the visual cortex (orange), but not the superior frontal cortex (green), contained information about the stimulus orientation (top right). However, on trials with a categorization demand, both the visual and superior frontal cortex showed representations of the stimulus (bottom right). Critically, on category trials, the superior frontal cortex was most strongly correlated with behavioral performance – participants did better when more stimulus information was available in this region.

As the participants went through the task, Shao et al. performed functional magnetic resonance imaging (fMRI) to measure patterns of activity across their entire brain. Data analysis aimed to capture, for each trial, how strongly the stimulus was represented in three brain areas: the early visual cortex, the intraparietal sulcus (a region important for orienting attention, high-level visual processing, and decision making), and the superior frontal cortex.

The results show that only one of the three areas, the frontal cortex, consistently held stronger stimulus representations during the more cognitively challenging categorization task, compared to the less demanding maintenance one (Figure 1). This difference suggests that the frontal cortex flexibly adapts its role during working memory as task demands and goals change. The strength of memory representations in the frontal cortex was also strongly correlated with how well participants performed in the categorization task but not the maintenance trials, further supporting the importance of this region in guiding goal-directed behavior.

Finally, the team constructed a recurrent neural network – a computational model inspired by the activity and connections between biological neurons – to solve a task similar to the one performed by the human participants. Built to mimic the three brain areas examined in the study, the network exhibited some flexibility across task demands, mirroring the activity detected in the frontal cortex via fMRI. Such similarity suggests that when trained to actively maintain future output in its ‘memory’, the network uses computations analogous to those unfolding in our own brains as working memory guides our future behavior. It remains a critical open question, though, how multiple brain (or neural network) areas operate together in real time to support working memory operations under different contexts.

The work by Shao et al. complements results from recent studies, which suggest that subtle changes in what we use our working memory for could explain why findings differ across brain areas and task contexts (Henderson et al., 2022; Shao et al., 2025). Yet, a kaleidoscope of additional factors related to stimuli, tasks, and learning regimes also likely contribute to patterns of flexible behavior and frontal cortex activity. For example, does the number, complexity and order of the rules that must be learned to complete the task affect working memory circuits? A person’s past learning experiences, and how these interact with long-term memory systems, may offer clues for understanding the many ways the frontal cortex supports flexible thinking and behavior (Bhandari et al., 2025; Miller et al., 2022). And finally, as always, we are also left to ponder what overarching ‘meta’ systems control which brain circuits and behaviors are recruited to accomplish the right goal in the correct context.
